# Prognostic value of the third thoracic vertebra skeletal muscle measurements in patients with digestive system malignancies: a comparative study with the third lumbar vertebra indices

**DOI:** 10.1038/s41598-026-37915-y

**Published:** 2026-01-30

**Authors:** Yuwei He, Yuguang Li, Yixin Zhao, Xinqiao Chen, Wei Ji, XiangLiang Liu, JiuWei Cui

**Affiliations:** 1 Center of Cancer, The First Hospital of Jilin University, Changchun, China; 2 Jilin University, Xinmin St No 71, Changchun, 130021 China

**Keywords:** Skeletal muscle mass, Computed tomography, Digestive system malignancy, Prognosis, Third thoracic vertebra, Third lumbar vertebra, Cancer imaging, Gastrointestinal cancer

## Abstract

Skeletal muscle mass assessment using computed tomography (CT) is crucial for evaluating nutritional status and prognosis in cancer patients. While the third lumbar vertebra (L3) level is widely accepted for this purpose, not all patients undergo abdominal CT scans. This study aimed to explore the potential of the third thoracic vertebra (T3) level as an alternative measurement site. This retrospective study included 257 patients with digestive system malignancies. Skeletal muscle area (SMA) and skeletal muscle index (SMI) were measured at both T3 and L3 levels using CT scans. Correlation analyses, linear regression models, and cox regression analyses were performed to evaluate the relationship between T3 and L3 measurements and their prognostic value. Strong correlations were observed between T3 and L3 measurements (*r* = 0.833 for SMA, *r* = 0.747 for SMI). A multivariate linear regression model effectively predicted L3 SMA from T3 SMA (adjusted *R*² = 0.829). Cox regression analyses revealed that lower T3 SMA and SMI were independently associated with increased mortality risk. Patients in the lowest quartile of T3 SMA had significantly higher mortality risk compared to those in the highest quartile (HR = 5.82, 95% CI: 1.86–18.16, *P* = 0.002), after adjusting for confounders. Similar results were observed for T3 SMI and L3 measurements. T3 skeletal muscle measurements strongly correlate with L3 measurements and serve as independent prognostic factors in patients with digestive system malignancies. T3 measurements offer a viable alternative for assessing skeletal muscle mass and predicting prognosis when L3 measurements are unavailable.

**Supplementary Information:**

The online version contains supplementary material available at 10.1038/s41598-026-37915-y.

## Introduction

Malignant tumors represent a unique group of chronic diseases that significantly impact patients’ body composition and metabolism throughout the disease course. The tumor microenvironment, systemic inflammation, metabolic alterations, and anti-cancer treatments can lead to accelerated skeletal muscle wasting in cancer patients^1,2^. This loss of muscle mass and function, known as sarcopenia, has been associated with decreased immune function, exacerbated inflammatory responses, reduced efficacy of anti-cancer therapies, and shortened survival^3–7^.

The prevalence of sarcopenia in cancer patients is alarmingly high, with studies reporting rates of 38.6% across all cancers and 44-57.7% specifically in digestive system malignancies^8–11^. Given its profound impact on patient outcomes, accurate assessment of skeletal muscle mass has become crucial in oncology for prognostication and treatment planning^12,13^.

Computed tomography (CT) is widely regarded as one of the most accurate methods for evaluating body composition, including skeletal muscle mass^14^. The third lumbar vertebra (L3) level has been established as the gold standard for assessing skeletal muscle area (SMA) and skeletal muscle index (SMI) in cancer patients^7,12,15^. However, not all cancer patients undergo abdominal CT scans at diagnosis, such as patients with lung cancer, esophageal cancer, and other thoracic tumors, potentially missing the opportunity for early muscle mass evaluation.

To address this limitation, researchers have begun exploring alternative anatomical levels for muscle assessment^12,15–17^. The third thoracic vertebra (T3) level, visible on chest CT scans, may potentially be a promising candidate due to its anatomical features and potential to reflect overall muscle mass. However, the relationship between T3 and L3 muscle measurements, as well as the prognostic value of T3-derived metrics in cancer patients, remains to be fully elucidated.

This study aims to investigate the correlation between skeletal muscle measurements at the T3 and L3 levels, develop a mathematical model for converting T3 to L3 measurements, and evaluate the prognostic significance of T3-derived muscle indices in patients with digestive system malignancies. By establishing T3 as a viable alternative to L3 for muscle mass assessment, we seek to expand the applicability of CT-based body composition analysis in oncology and improve risk stratification for patients lacking abdominal CT scans.

## Patients and methods

### Study design and population

This retrospective study included patients diagnosed with digestive system malignancies at the Cancer Center of the First Hospital of Jilin University between July 2013 and December 2018 and was approved by the Ethics Committee of the first affiliated hospital of Jilin University (2017–362). All study participants have filled out written informed consent for participation and this research complied with the Declaration of Helsinki.

Patients are screened through a rigorous process. The inclusion criteria of patients included: (1) Patients older than 18; (2) Patients with histologically confirmed diagnosis of digestive system malignancy; (3) Availability of abdominal and chest CT scans within a one-month window. And the exclusion criteria included: (1) Patients with multiple primary malignancies; (2) Patients without critical data or incomplete records; (3) Patients with incomplete CT images.

**Fig. 1:**
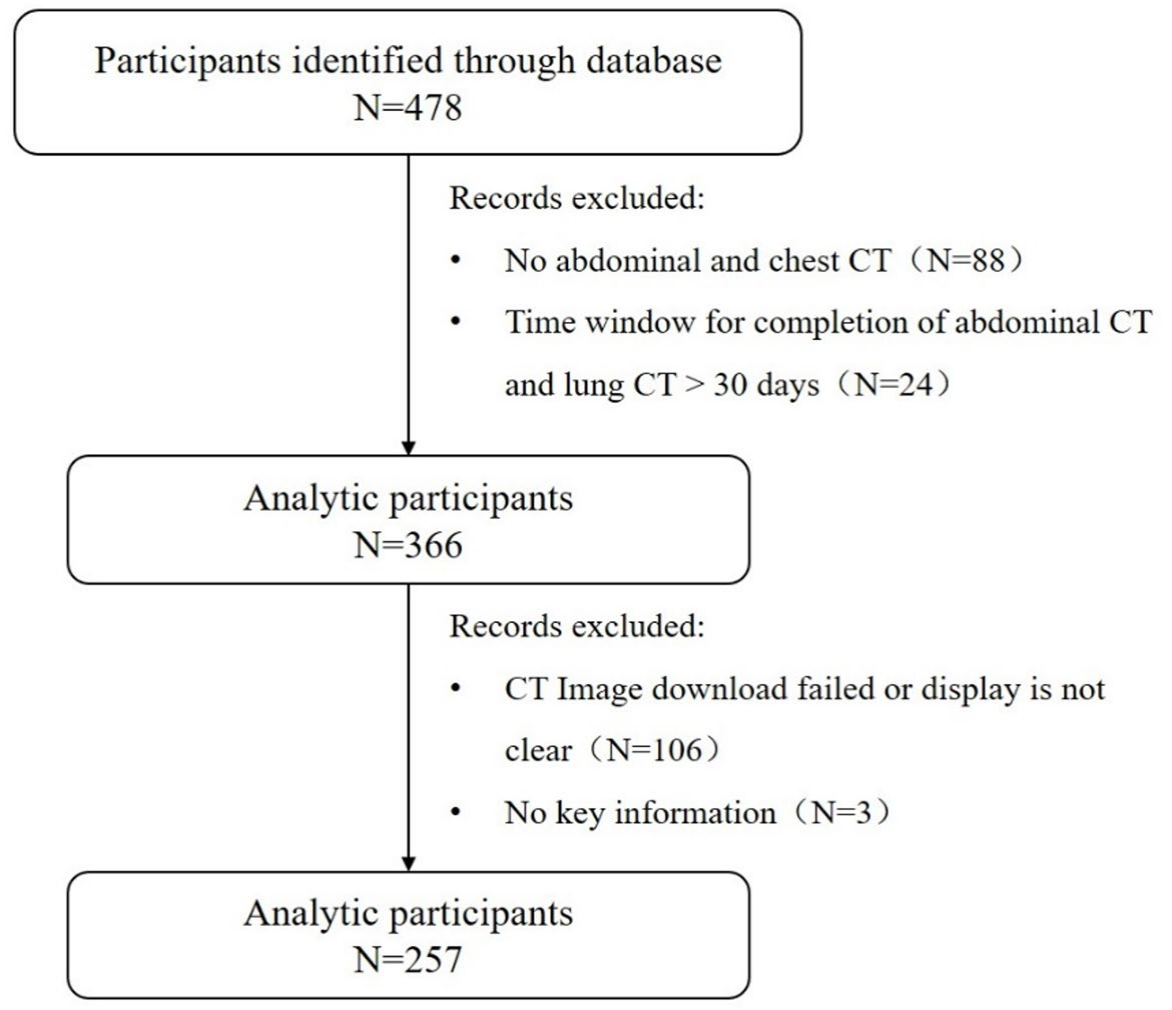
Study flow chart.

In this research, 478 patients were eventually collected, of which 336 after exclusion of patients lacking abdominal or chest CT and patients without two CTs completed < 30 days. Patients who had not key information and patients with problematic CT images were excluded and 257 were included in the final analysis (Fig. 1).

### Data collection

Clinical data collected included demographic characteristics (age and sex), anthropometric measurements (height and weight), and laboratory parameters. Height and weight were measured with patients wearing light clothing and no shoes, after a 2-hour fast and bladder emptying. Body Mass Index (BMI) was calculated as weight/height² (kg/m²). Cancer stages were evaluated based on the 8th edition of the American Joint Committee on Cancer (AJCC) TNM staging system.

### Laboratory information

Fasting venous blood samples were collected on the second morning of first admission. Parameters recorded included peripheral lymphocyte count, neutrophil count, platelet count, albumin (ALB, (g/L)), and high-sensitivity C-reactive protein (CRP, (mg/L)). Neutrophil-to-lymphocyte ratio (NLR) and platelet-to-lymphocyte ratio (PLR) were calculated. Prognostic nutritional index (PNI) was calculated as follows: PNI = albumin (g/L) + 5 × lymphocyte count (×10^9^/L).

### Study endpoint

The primary endpoint was overall survival (OS), defined as the time from cancer diagnosis to death from any cause. Patients alive at the last follow-up (January 1st, 2020) were censored.

### Computed tomography and body composition analysis

**Fig. 2:**
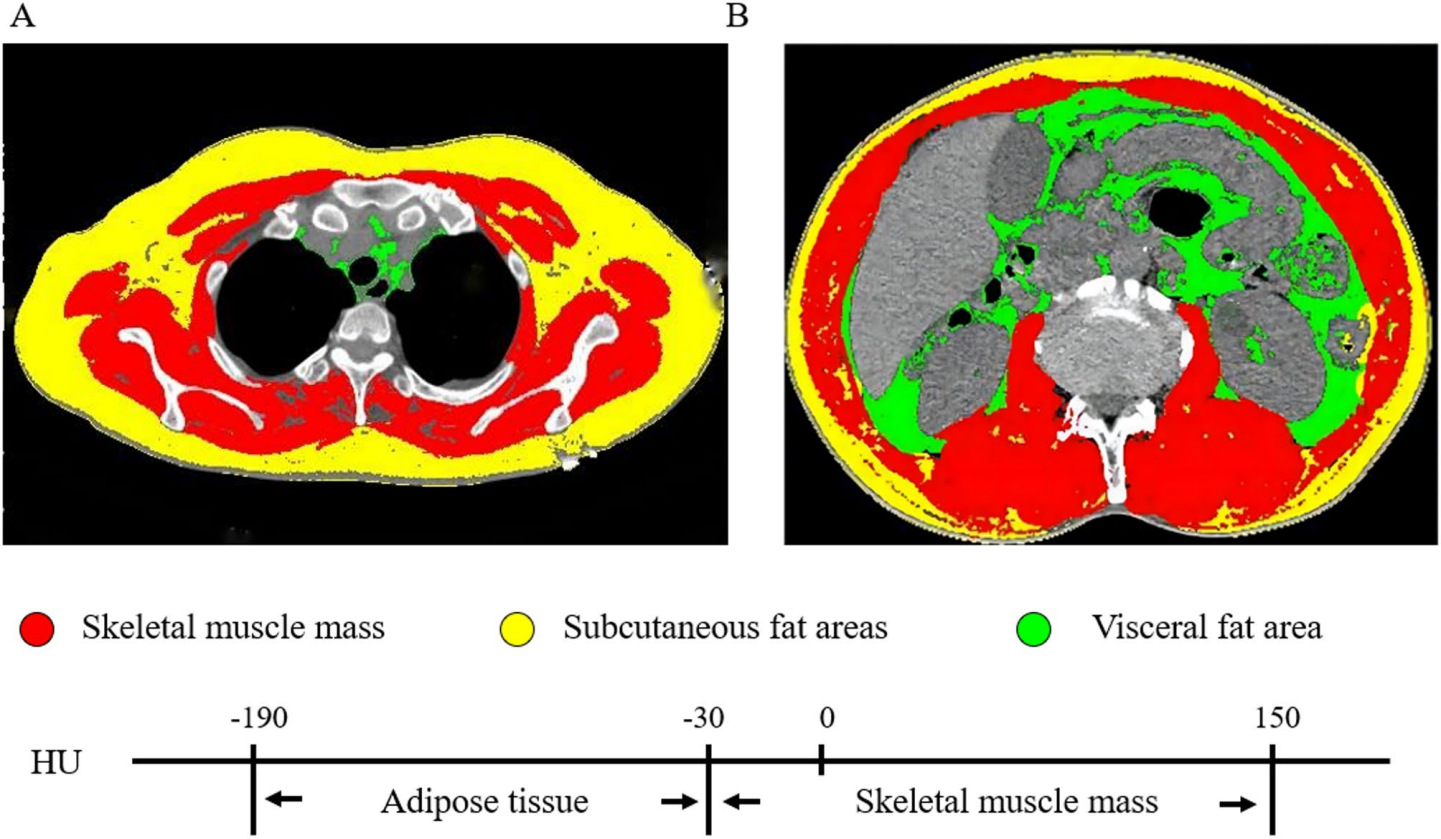
The skeletal muscle mass and adipose tissue at the third thoracic vertebrae level (**A**) and the third lumbar vertebra level (**B**).

Collect chest and abdominal CT imaging data from all patients initially diagnosed with digestive system malignancies by pathological examination. All images were acquired using the following spiral CT scanners: PHILIPS Ict64/256, SIEMENS Cardiac 64, and GE Revolution 64. After scanning, all images were uniformly reconstructed into 5 mm-thick slices and downloaded in DICOM format. The plane at the T3 level was defined by identifying the T3 rib and ensuring full exposure of the corresponding spinous process. Similarly, the plane at the L3 level was established by locating the complete spinous process at the same vertebral level. CT images were sequentially analyzed using SliceOmatic 5.0 software (Tomovision, Canada). Skeletal muscle area (SMA) were measured at L3 and T3 levels. Tissue was identified using Hounsfield unit (HU) thresholds: -29 to 150 HU for skeletal muscle. Skeletal muscle index (SMI) was calculated as SMA/height² (cm²/m²) (Fig. 2)^18,19^.

### Statistical analysis

Data analysis was performed using SPSS 26.0 and R version 4.0.5. Normality was assessed using the Shapiro-Wilk test. Continuous variables were presented as mean ± standard deviation (Mean ± SD) or median (interquartile range) (Median ( IQR)), and categorical variables as count (percentage)[N(%)]. Spearman’s correlation analysis was utilized to assess relationships between CT measurements and laboratory parameters. Linear regression models were developed to predict L3 SMA from T3 SMA. Bland-Altman plots were used to assess agreement between predicted and actual L3 SMA values. The computed SMA and SMI at the T3 and L3 levels were grouped into quartiles, defined from largest to smallest as groups Q1, Q2, Q3, and Q4, respectively. Cox proportional hazards models and Kaplan-Meier(K-M) survival curves were used to evaluate associations between muscle groups above and overall survival, and the P-value for the comparison between the two groups was calculated by the Log-rank test. Hazard ratios (HRs) and their 95% confidence intervals (CIs) were calculated after cox proportional hazards models. The multivariable cox proportional hazards models were adjusted for sex, age, stage and cancer types. Restricted cubic spline (RCS) analyses were conducted to explore non-linear relationships adjusting for sex, age, stage and cancer type. Interaction terms were used to assess the confounding effects of age and sex. A bilateral P-value < 0.05 was considered statistically significant.

## Results

### Patient characteristics

Of 257 patients eventually screened, the cohort comprised 161 males (62.6%) and 96 females (37.4%). The most common cancer types were colorectal (50.2%), gastric (21.0%), and pancreatic (7.7%). Stage III disease was present in 101 patients (39.2%), and stage IV in 60 patients (23.3%). Mean T3 SMA was 238.31 ± 34.81 cm² for males and 155.07 ± 26.90 cm² for females. Mean L3 SMA was 145.13 ± 26.14 cm² for males and 93.57 ± 17.24 cm² for females. T3 SMI averaged 81.85 ± 12.28 cm²/m² for males and 62.75 ± 11.01 cm²/m² for females, while L3 SMI averaged 49.81 ± 8.81 cm²/m² for males and 37.78 ± 6.54 cm²/m² for females (Table 1).

**Table 1:** Basic characteristics of patients.

Characteristics	Overall(*N* = 257) Mean ± SD/*n* (%)
Age, years	58.80 ± 10.19
< 65	190(73.9)
≥ 65	67(26.1)
Sex, n(%)	
Male	161(62.6)
Female	96(37.4)
Height (cm)	167(13)
Weight (kg)	62.19 ± 11.60
BMI	22.55 ± 3.42
Diabetes	
Yes	35(13.6)
No	222(86.4)
Hypertension	
Yes	52(20.2)
No	205(79.8)
Cancer type	
Gastric cancer	54(21.0)
Liver cancer	15(5.8)
Esophagus cancer	12(4.6)
Pancreatic cancer	20(7.7)
Colorectal cancer	142(50.2)
Biliary tract carcinoma	12(4.6)
Duodenal carcinoma	1(0.35)
Gallbladder carcinoma	1(0.35)
TNM stage	
I	19(7.3)
II	77(29.9)
III	101(39.2)
IV	60(23.3)
T3 SMA (cm^2^)	
Male	238.31 ± 34.81
Female	155.07 ± 26.90
L3 SMA (cm^2^)	
Male	145.13 ± 26.14
Female	93.57 ± 17.24
T3 SMI (cm^2^/m^2^)	
Male	81.85 ± 12.28
Female	62.75 ± 11.01
L3 SMI (cm^2^/m^2^)	
Male	49.81 ± 8.81
Female	37.78 ± 6.54

BMI: body mass index; SMA: skeletal muscle area; SMI: skeletal muscle index.

### Correlation of T3 and L3 measurements

As showed in Table 2, Strong correlations were observed between T3 and L3 measurements. Overall, SMA showed a strong correlation (*r* = 0.833) and SMI a good correlation (*r* = 0.747) between T3 and L3 levels. These correlations remained strong across age and stage subgroups.

**Table 2:** Spearman correlation analysis of different indicates between the third thoracic vertebrae level and the third lumbar vertebra level.

		SMA		SMI	
	*N*	*r*	*P*	*r*	*P*
Overall	257	0.833	< 0.001	0.747	< 0.001
Sex, n(%)					
Male	161	0.589	< 0.001	0.599	< 0.001
Female	96	0.639	< 0.001	0.583	< 0.001
Age, years					
< 65	190	0.816	< 0.001	0.719	< 0.001
≥ 65	67	0.871	< 0.001	0.828	< 0.001
TNM stage					
I-III	197	0.846	< 0.001	0.760	< 0.001
IV	60	0.826	< 0.001	0.744	< 0.001

SMA: skeletal muscle area; SMI: skeletal muscle index.

### Relationship of laboratory parameters and muscle indices

T3 and L3 muscle measurements showed weak to moderate correlations with inflammatory markers (Table S1). Notably, albumin and PNI positively correlated with all muscle indices, while CRP negatively correlated with T3 SMA and SMI.

### Prediction model for L3 SMA

**Fig. 3:**
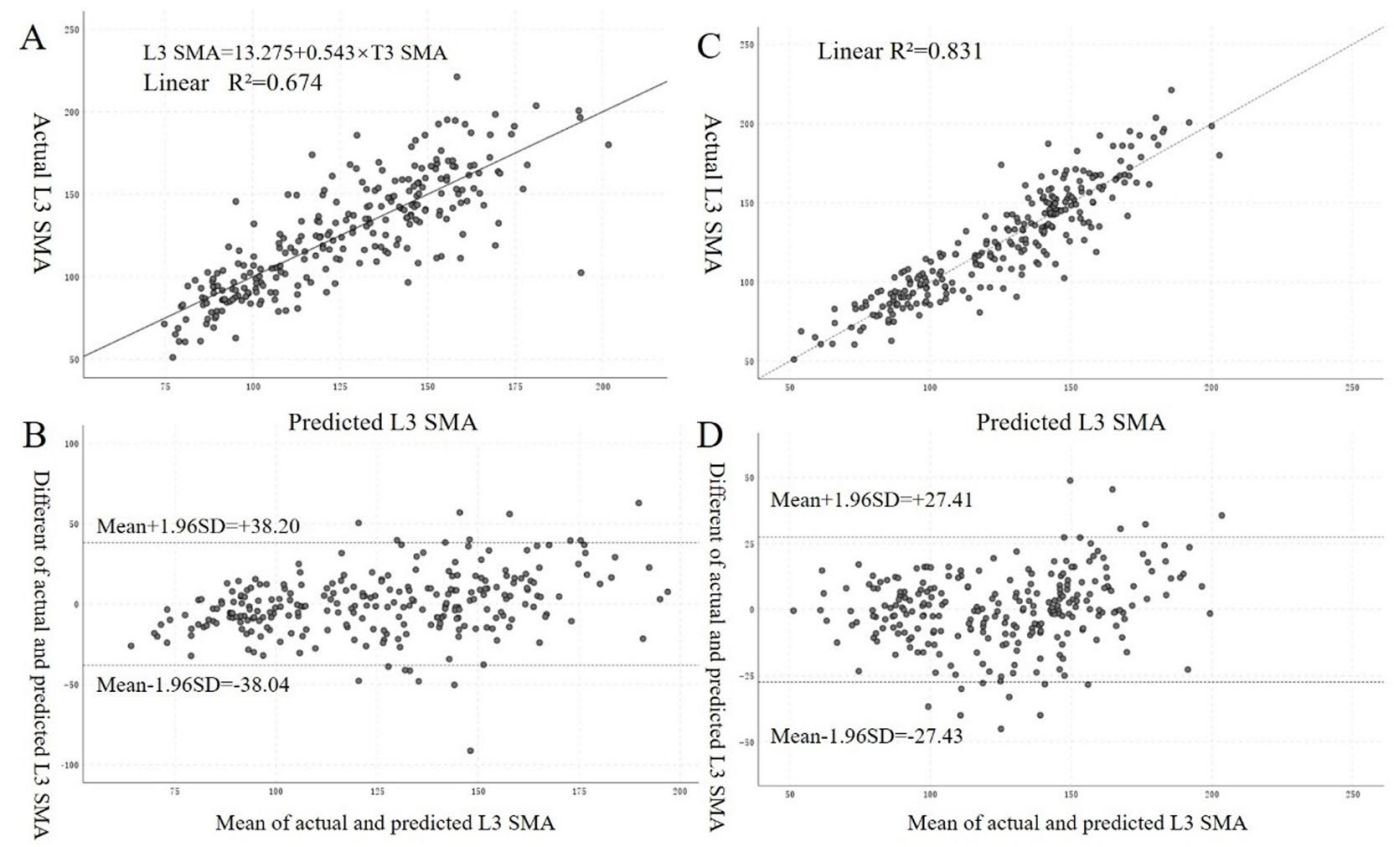
Formulas establish and verify by using univariable (**A** and **B**) and multivariable (**C** and **D**) linear regression analysis. The formulates obtained from univariable and multivariable regression analysis were applied for consistency validation between predicted L3 SMA and actual L3 SMA. (**A**) Scatter plot showed significant correlation between predicted L3 SMA and actual L3 SMA in the univariable regression model. (**B**) Bland-Altman plot showed the consistency between the predicted L3 SMA and the actual L3 SMA in the univariable regression model, with the difference between the measurements shown on the y-axis and the mean shown on the x-axis. (**C**) Scatter plot showed significant correlation between predicted L3 SMA and actual L3 SMA in the multivariable regression model. (**D**) Bland-Altman plot showed the agreement between the predicted L3 SMA and the actual L3 SMA in the multivariable regression model, with the difference between measurements shown on the y-axis and the mean shown on the x-axis.

A multivariate linear regression model was developed to predict L3 SMA: L3 SMA = 32.380 + 0.272 × T3 SMA − 13.979 × Sex + 1.250 × Weight − 0.364 × Age (male = 1, female = 2) (adjusted *R*² = 0.829, *P* < 0.001), whose goodness of fit index was better than univariate regression analysis (adjusted *R*² = 0.673, *P* < 0.001). Bland-Altman plot also demonstrated good agreement between predicted and actual L3 SMA values (Fig. 3). All relevant parameters were displayed in Table S2.

### Association with overall survival

**Fig. 4:**
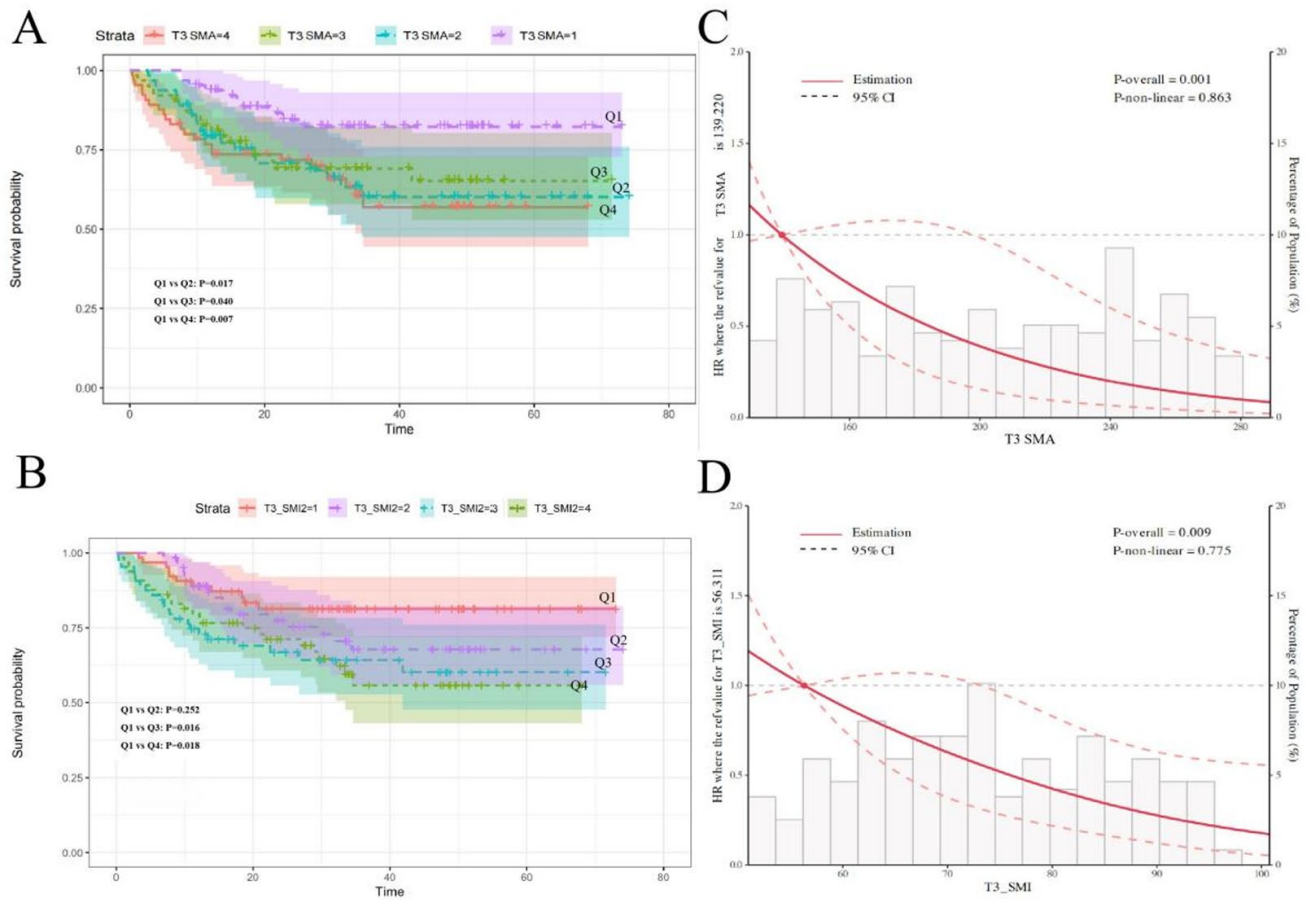
The correlation between T3 SMA and T3 SMI quartile group and prognosis via Kaplan-Meier (K-M) curves (**A** and **B**) and restricted cubic spline (RCS) plots (**C** and **D**). RCS plots were analyzed after adjusting of sex, age, stage and cancer types.

**Fig. 5:**
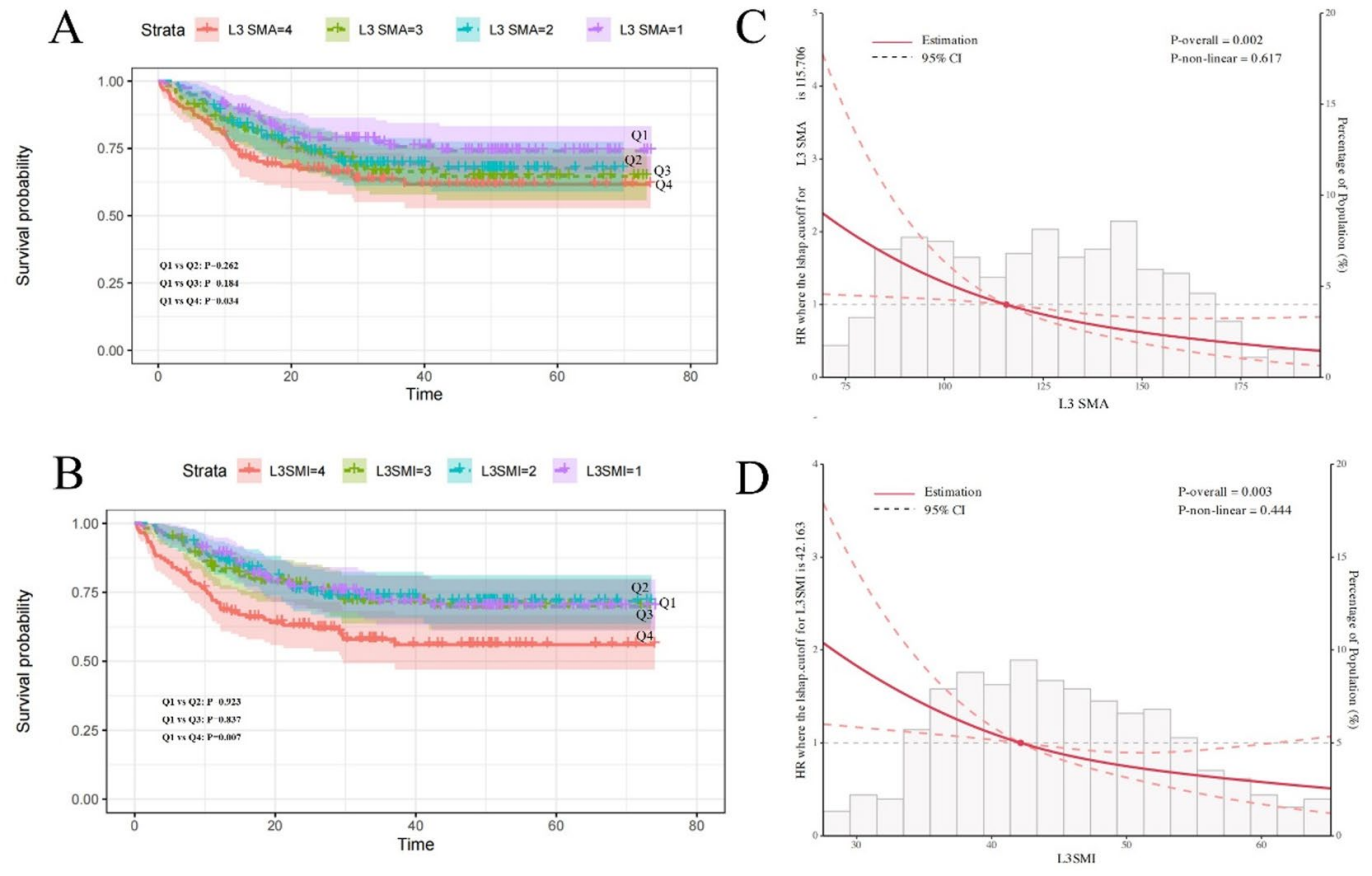
The correlation between L3 SMA and L3 SMI quartile group and prognosis via Kaplan-Meier (K-M) curves (**A** and** B**) and restricted cubic spline (RCS) plots (**C** and** D**). RCS plots were analyzed after adjusting of sex, age, stage and cancer types.

Cox regression analysis revealed that lower T3 SMA and T3 SMI were associated with increased mortality risk (Table 3). When stratified by T3 SMA quartiles, patients in the lowest quartile (Q4) had significantly higher mortality risk compared to those in the highest quartile (Q1) (HR = 5.82, 95% CI: 1.86–18.16, *P* = 0.002), after adjusting for age, sex, stage, and cancer type. Similar results were observed for T3 SMI, with the lowest quartile(Q4) showing increased mortality risk (HR = 3.97, 95% CI: 1.54–10.26, *P* = 0.004) compared to the highest quartile(Q1). Kaplan-Meier survival curves demonstrated significant differences in overall survival across T3 SMA and SMI quartiles (Fig. 4A-B). Restricted cubic spline (RCS) analysis revealed a linear relationship between T3 muscle metrics and mortality risk. The risk of death increased sharply when T3 SMA fell below approximately 139 cm² (*P* = 0.001) and when T3 SMI was below 56 cm²/m² (*P* = 0.009) (Fig. 4C-D). L3 measurements showed similar prognostic value, with lower L3 SMA and SMI associated with increased mortality risk (Table 4; Fig. 5). Age, but not sex, significantly modified the effect of muscle measurements on survival (Table S3, Table S4). These findings suggest that both T3 and L3 skeletal muscle measurements serve as independent prognostic factors in patients with digestive system malignancies, with T3 measurements offering a viable alternative when L3 measurements are unavailable.

**Table 3:** The correlation between T3 SMA/SMI and prognosis by using univariable and multivariable regression analysis.

	Univariable		Multivariable*	
	HR (95%CI)	*P*	HR (95%CI)	*P*
T3 SMA				
Q1	Reference		Reference	
Q2	2.50(1.18, 5.32)	0.017	2.91(1.31, 6.46)	0.009
Q3	2.23(1.04, 4.80)	0.040	3.88(1.65, 9.14)	0.002
Q4	2.77(1.33, 5.80)	0.007	5.82(1.86, 18.16)	0.002
T3 SMI				
Q1	Reference		Reference	
Q2	1.56(0.73, 3.33)	0.252	1.28(0.56, 2.94)	0.558
Q3	2.44(1.18, 5.03)	0.016	3.33(1.45, 7.63)	0.004
Q4	2.37(1.16, 4.85)	0.018	3.97(1.54, 10.26)	0.004

*Multivariable models were analyzed after adjusting of sex, age, stage and cancer types.

**Table 4:** The correlation between L3 SMA/SMI and prognosis by using univariable and multivariable regression analysis.

	Univariable		Multivariable*	
	HR (95%CI)	*P*	HR (95%CI)	*P*
L3 SMA				
Q1	Reference		Reference	
Q2	1.95(0.97, 3.95)	0.062	2.66(1.26, 5.63)	0.010
Q3	1.63(0.79, 3.36)	0.184	2.72(1.14, 6.50)	0.025
Q4	2.16(1.06, 4.38)	0.034	3.33(1.17, 9.49)	0.024
L3 SMI				
Q1	Reference		Reference	
Q2	0.98(0.59, 1.61)	0.923	1.18(0.69, 2.02)	0.537
Q3	1.05(0.64, 1.73)	0.837	1.57(0.91, 2.7)	0.105
Q4	1.86(1.18, 2.92)	0.007	3.10(1.71, 5.61)	< 0.001

*Multivariable models were analyzed after adjusting of sex, age, stage and cancer types.

In subgroup K-M analyses of colorectal and gastric cancers, Figure S1 demonstrates a correlation between skeletal muscle mass of T3 vertebra and risk of death in patients with colorectal cancer, albeit with a P-value of greater than 0.05. At the same time, in patients with gastric cancer, regardless of plane, it demonstrates that a higher SMA or SMI is associated with a lower risk of death (Figure S2).

## Discussion

This study demonstrates a strong correlation between skeletal muscle measurements at the T3 and L3 levels in patients with digestive system malignancies, establishing T3 as a viable alternative for muscle mass assessment when abdominal CT scans are unavailable. Our findings not only validate the use of T3 measurements but also highlight their prognostic significance, potentially expanding the scope of sarcopenia evaluation in oncology.

Our results clearly demonstrate a strong correlation between skeletal muscle mass at the T3 and L3 vertebral levels. The robust correlation observed between T3 SMA and L3 SMA (*r* = 0.833) aligns with previous research exploring alternative anatomical levels for muscle assessment^16,20^. This correlation remained consistent across different age groups and cancer stages, indicating the reliability of T3 measurements across diverse patient populations. The slightly lower correlation for SMI (*r* = 0.747) may be attributed to the influence of height in its calculation, introducing additional variability. In digestive system malignancies, we can readily obtain concurrent lung and abdominal imaging, enabling validation of the T3-L3 correlation. This approach establishes a foundation for assessing muscle mass in treatment-naïve patients with thoracic malignancies (e.g., lung cancer, esophageal cancer, and other chest tumors) who typically undergo chest CT without abdominal imaging.

The strong relationship between T3 and L3 measurements can be explained by several physiological and anatomical factors. Skeletal muscle mass distribution follows a relatively consistent pattern across individuals, with proportional changes occurring throughout the body in response to various stimuli^21^. The T3 level captures major muscle groups such as the pectoralis major, trapezius, and rhomboids, which are representative of upper body musculature. Similarly, the L3 level includes key core and lower body muscles like the psoas, erector spinae, and quadratus lumborum^22^. Both levels reflect overall muscle status, contributing to their high correlation.

Moreover, cancer-related muscle wasting tends to affect multiple muscle groups simultaneously due to systemic factors such as inflammation, metabolic dysregulation, and reduced physical activity^23^. This generalized effect on muscle mass likely enhances the correlation between T3 and L3 measurements. Additionally, both levels are relatively stable anatomical landmarks, minimally affected by respiratory motion or postural changes, further improving their reliability for muscle assessment.

Our multivariate linear regression model for predicting L3 SMA from T3 SMA demonstrated excellent fit (adjusted *R*^2^ = 0.829), outperforming the univariate model. According to previous studies, the inclusion of age, sex, and body weight as covariates improved the model’s accuracy, highlighting the importance of considering these factors in muscle mass estimation^24,25^. The Bland-Altman plot analysis further confirmed the good agreement between predicted and actual L3 SMA values, supporting the clinical applicability of our conversion formula.

Importantly, we found that both T3 and L3 muscle measurements were significantly associated with overall survival in digestive system malignancy patients. Lower T3 SMA and SMI were linked to increased mortality risk, consistent with the established prognostic value of L3 measurements^1,26,27^. The RCS analysis revealed a non-linear relationship between muscle mass and survival, with risk increasing sharply below certain thresholds. These findings underscore the potential of T3 measurements for risk stratification and treatment planning in oncology identification of cancer patients with potential sarcopenia. Precise evaluation of skeletal muscle status carries profound implications for comprehensive patient management and prognostic prediction.

The use of T3 level measurements offers several advantages. Chest CT scans are routinely performed for diagnosis and staging in many cancer types, providing an opportunity for muscle assessment without additional imaging^28–30^. The T3 level is less affected by bowel distension or ascites, which can impact L3 level assessments in some patients. Furthermore, utilizing T3 for sarcopenia evaluation expands the pool of patients who can undergo muscle mass assessment, potentially leading to earlier detection of sarcopenia and timely interventions.

The significance of T3-based evaluation extends beyond convenience. It facilitates more comprehensive longitudinal monitoring of muscle changes throughout cancer treatment, as regular chest CT scans are often performed to assess treatment response in many cancer types. This allows for concurrent tracking of muscle mass without additional radiation exposure. Moreover, the validation of T3 measurements opens new avenues for research into cancer-related sarcopenia, enabling retrospective studies using existing chest CT databases and potentially accelerating our understanding of muscle wasting patterns across various cancer types and stages. Additionally, supportive care adjustments will be implemented, including: nutritional education on dietary structure, optimal timing for enteral/parenteral nutrition, immunonutrient supplementation, caloric intake modification, and physical activity guidance. The current priority strategies for cancer patient management involve: (1) rapid identification and assessment of low muscle mass, (2) developing management protocols for at-risk patients, and (3) nutritional optimization to enhance recovery^31^.

From a clinical perspective, incorporating T3 muscle assessment into routine practice could enhance risk stratification and guide personalized treatment decisions. Identifying patients with low muscle mass using T3 measurements could inform decisions about treatment intensity, nutritional interventions, or prehabilitation strategies. The broader implementation of T3-based sarcopenia evaluation also has potential implications for standardizing muscle assessment in oncology, promoting greater consistency in research and clinical practice.

### Strengths and limitations

Our study demonstrates several key strengths. We employed robust statistical methods to validate T3 measurements against the established L3 standard, using a relatively large sample of 257 digestive system malignancy patients. Beyond establishing correlations, we demonstrated the prognostic significance of T3 measurements and provided a practical conversion formula for estimating L3 SMA from T3 SMA. The consistency of our findings across different subgroups supports the broad applicability of T3 measurements. Importantly, our study addresses a critical gap by providing evidence for an alternative anatomical level for muscle mass assessment, potentially expanding sarcopenia evaluation to patients without available abdominal CT scans. These strengths collectively contribute to the robustness and clinical relevance of our findings, laying a foundation for future applications of T3-based muscle mass assessment in oncology.

However, some limitations should be acknowledged. As a single-center, retrospective study focusing on digestive system malignancies, our findings may not be fully generalizable to all cancer types or populations. Future multi-center studies with larger, more diverse cohorts are needed to confirm and extend our results. Additionally, our analysis was based on baseline CT scans and did not account for changes in muscle mass over time. Longitudinal studies assessing the prognostic value of muscle loss trajectories using T3 measurements could provide further insights into the dynamics of cancer-related sarcopenia^32^. Furthermore, we acknowledge that the inclusion of patients exclusively with digestive system malignancies introduces inconsistent standards in TNM staging across different cancer types and subtypes. This variability may confound the observed relationships between muscle mass and clinical outcomes. Future studies with larger cohorts may help mitigate this limitation. In addition, the manual segmentation of CT images in our study, while cross-checked, may introduce some subjectivity. The development of automated, artificial intelligence-based segmentation tools could improve accuracy and efficiency in future research and clinical applications^28,31^.

## Conclusion

In conclusion, our study establishes the validity and prognostic value of T3-level skeletal muscle measurements in digestive system malignancy patients. These findings pave the way for more comprehensive and widespread assessment of sarcopenia in oncology, potentially benefiting patients for whom abdominal CT scans are unavailable. Future research should focus on refining prediction models, exploring muscle quality metrics, and integrating T3 measurements into clinical decision-making algorithms. As we continue to refine and implement this method, it holds promise for improving outcomes and quality of life for cancer patients across the globe.

## Supplementary Information

Below is the link to the electronic supplementary material.

Supplementary Material 1

## Data Availability

The authors declare that all supporting data are available within the article and the online supplementary files. Because of the sensitive nature of the data collected for this study, requests to access the data set may be sent to the corresponding author.

## Abbreviations

CT: Computed tomography
T3: The third thoracic vertebra
L3: The third lumbar vertebra
SMA: Skeletal muscle area
SMI: Skeletal muscle index
BMI: Body mass index
AJCC: American joint committee on cancer
ALB: Albumin
CRP: C-reactive protein
NLR: Neutrophil-to-lymphocyte ratio
PLR: Platelet-to-lymphocyte ratio
OS: Overall survival
SMA: Skeletal muscle area
HU: Hounsfield unit
SMI: Skeletal muscle index
K-M: Kaplan-Meier
HRs: Hazard ratios
CIs: Confidence intervals
RCS: Restricted cubic spline
