# Supplementary material for: Prognostic value of the third thoracic vertebra skeletal muscle measurements in patients with digestive system malignancies: a comparative study with the third lumbar vertebra indices
Source: Sci Rep. 2026 Jan 30;16:6749. doi: 10.1038/s41598-026-37915-y (PMC12913669; doi:10.1038/s41598-026-37915-y)
Supplement: Supplementary file 1 — Supplementary Material 1 [file 41598_2026_37915_MOESM1_ESM.docx]

Supplemental Table 1：Spearman correlation analysis between different muscle indicates and laboratory index.

|  | L3 SMA | |  | T3 SMA | |  | L3 SMI | |  | T3 SMI | |
| --- | --- | --- | --- | --- | --- | --- | --- | --- | --- | --- | --- |
|  | *r* | *P* |  | *r* | *P* |  | *r* | *P* |  | *r* | *P* |
| L3 SMA | 1 | <0.001 |  | 0.833 | <0.001 |  | -0.941 | <0.001 |  | 0.728 | <0.001 |
| T3 SMA | 0.833 | <0.001 |  | 1 | <0.001 |  | 0.742 | <0.001 |  | 0.930 | <0.001 |
| L3 SMI | 0.941 | <0.001 |  | 0.742 | <0.001 |  | 1 | <0.001 |  | 0.747 | <0.001 |
| T3 SMI | 0.728 | <0.001 |  | 0.930 | <0.001 |  | 0.747 | <0.001 |  | 1 | <0.001 |
| CRP | -0.123 | 0.128 |  | -0.172 | 0.033 |  | -0.130 | 0.108 |  | -0.189 | 0.019 |
| ALB | 0.181 | 0.004 |  | 0.169 | 0.007 |  | 0.193 | 0.002 |  | 0.173 | 0.005 |
| NLR | -0.131 | 0.036 |  | -0.084 | 0.181 |  | -0.126 | 0.044 |  | -0.061 | 0.328 |
| PLR | -0.202 | 0.001 |  | -0.144 | 0.021 |  | -0.181 | 0.004 |  | -0.113 | 0.072 |
| PNI | 0.185 | 0.003 |  | 0.152 | 0.015 |  | 0.200 | 0.001 |  | 0.154 | 0.013 |

SMA: skeletal muscle area; SMI: skeletal muscle insex; CRP:C-reactive protein；ALB: albumin; NLR: neutrophil to lymphocyte ratio; PLR: platelet to lymphocyte ratio; PNI: prognostic nutritional index.

Supplemental Table 2: The univariable and multivariable linear regression model of L3 SMA.

|  | *B* | *β* | *P* | *F* | Adjusted *R^2^* |
| --- | --- | --- | --- | --- | --- |
| Univariable model |  |  |  |  |  |
| T3 SMA | 0.543 (0.497, 23.215) | 0.821 | <0.001 | 528.220 | 0.673 |
| Constant | 13.275 (3.334, 23.215) |  | 0.009 |  |  |
| Multivariable model |  |  |  |  |  |
| Sex | -13.979 (-19.8, -7.807) | -0.199 | <0.001 | 310.843 | 0.829 |
| Weight | 1.250 (1.069, 1.431) | 0.426 | <0.001 |  |  |
| Age | -0.364 (-0.529, -0.170) | -0.109 | 0.001 |  |  |
| T3 SMA | 0.272 (0.214, 0.333) | 0.411 | <0.001 |  |  |
| Constant | 32.380 (5.032, 57.028) |  | 0.015 |  |  |

Supplemental Table 3: Interaction of age and gender in the relationship between T3 SMA/SMI and risk of death.

| Variants | N=257  n (%) | HR (95%CI) | *P* | *P after interaction* |
| --- | --- | --- | --- | --- |
| **T3 SMA** |  |  |  |  |
| Overall |  | 0.99(0.99, 1.00) | 0.005 |  |
| Age, years |  |  |  | 0.01 |
| <65 | 190(73.9) | 0.99(0.98, 1.00) | <0.001 |  |
| ≥65 | 67(26.1) | 1.00(1.00, 1.01) | 0.344 |  |
| Sex |  |  |  | 0.207 |
| Male | 161(62.6) | 0.99(0.98, 1.00) | 0.014 |  |
| Female | 96(37.4) | 0.98(0.96, 1.00) | 0.016 |  |
| **T3 SMI** |  |  |  |  |
| Overall |  | 0.98(0.99, 0.99) | 0.006 |  |
| Age, years |  |  |  | 0.005 |
| <65 | 190(73.9) | 0.97(0.95, 0.98) | <0.001 |  |
| ≥65 | 67(26.1) | 1.02(0.99, 1.05) | 0.242 |  |
| Sex |  |  |  | 0.312 |
| Male | 161(62.6) | 0.98(0.95, 1.00) | 0.058 |  |
| Female | 96(37.4) | 0.95(0.91, 0.99) | 0.026 |  |

Supplemental Table 4：Interaction of age and gender in the relationship between L3 SMA/SMI and risk of death.

| Variants | N=257  n (%) | HR (95%CI) | *P* | *P after interaction* |
| --- | --- | --- | --- | --- |
| **L3 SMA** |  |  |  |  |
| Overall |  | 0.99(0.99, 1.00) | 0.04 |  |
| Age, years |  |  |  | 0.003 |
| <65 | 190(73.9) | 0.99(0.98, 1.00) | 0.002 |  |
| ≥65 | 67(26.1) | 1.00(1.00, 1.03) | 0.115 |  |
| Sex |  |  |  | 0.848 |
| Male | 161(62.6) | 0.99(0.98, 1.00) | 0.088 |  |
| Female | 96(37.4) | 0.99(0.97, 1.01) | 0.285 |  |
| **L3 SMI** |  |  |  |  |
| Overall |  | 0.98(0.95, 1.00) | 0.065 |  |
| Age, years |  |  |  | 0.001 |
| <65 | 190(73.9) | 0.95(0.92, 0.98) | 0.002 |  |
| ≥65 | 67(26.1) | 1.05(1.00, 1.10) | 0.061 |  |
| Sex |  |  |  | 0.788 |
| Male | 161(62.6) | 0.98(0.94, 1.01) | 0.213 |  |
| Female | 96(37.4) | 0.97(0.92, 1.03) | 0.308 |  |

**Supplemental Figure 1**：The correlation between L3 SMA and L3 SMI **(A and B)**, T3 SMA and T3 SMI **(C and D)** quartile group and prognosis via Kaplan-Meier (K-M) curves in patients with colorectal cancer.


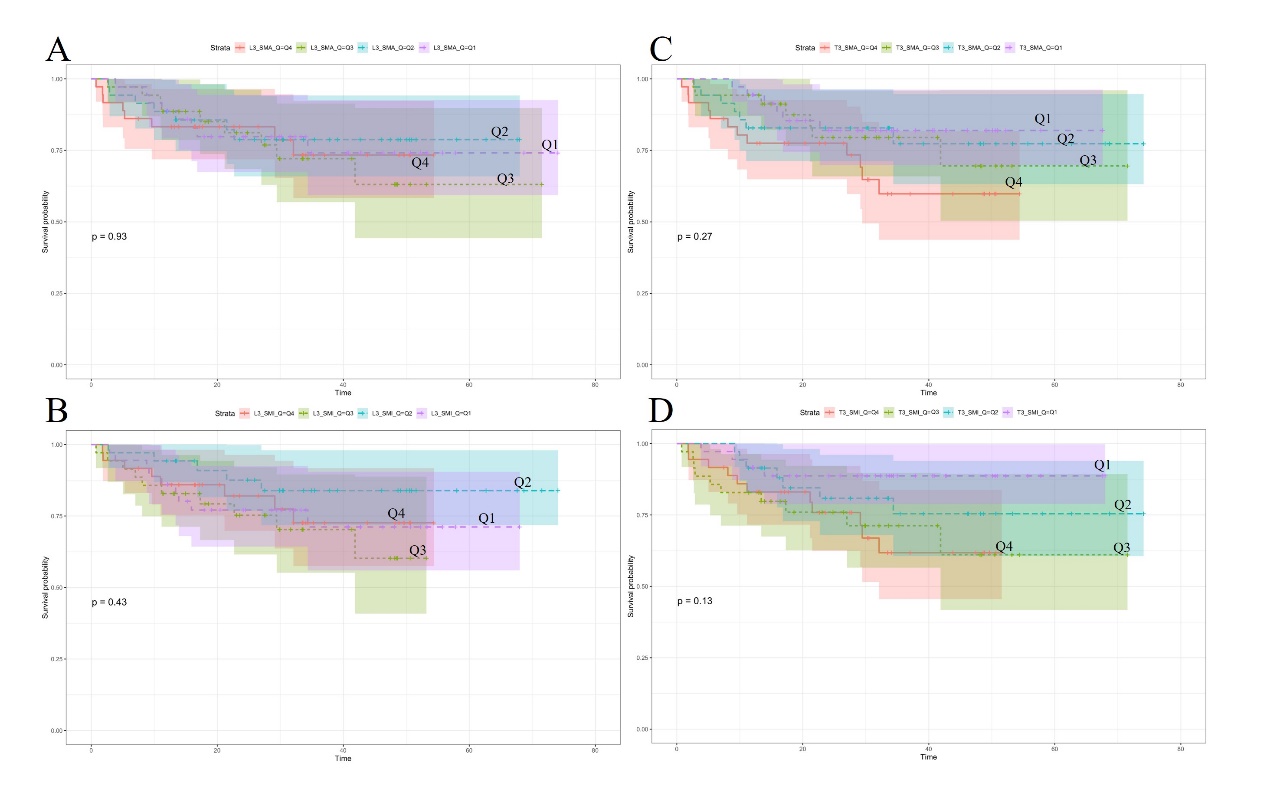


The log-rank test was used to compare the differences between the survival curves. A p-value less than 0.05 usually indicates a significant difference between the groups.

**Supplemental Figure 2**：The correlation between L3 SMA and L3 SMI **(A and B)**, T3 SMA and T3 SMI **(C and D)** tertile group and prognosis via Kaplan-Meier (K-M) curves in patients with gastric cancer.


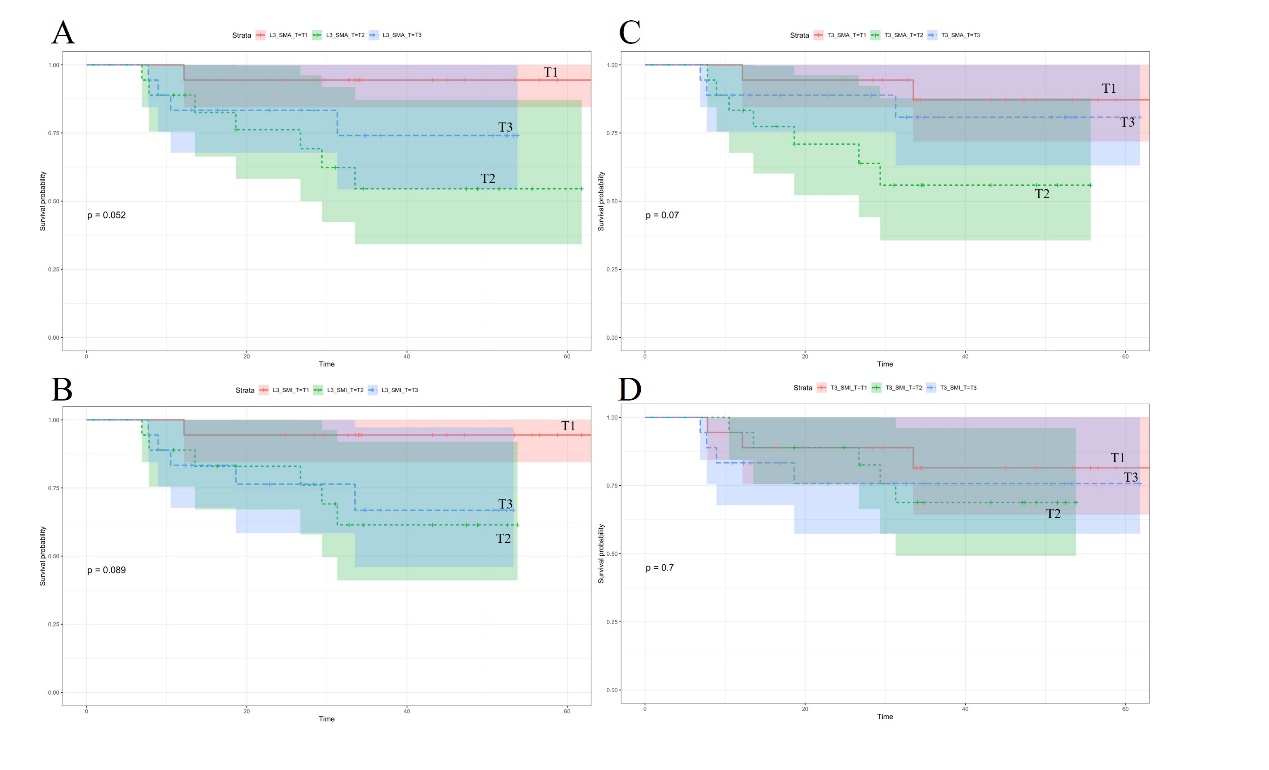


The log-rank test was used to compare the differences between the survival curves. A p-value less than 0.05 usually indicates a significant difference between the groups.
